# Zebrafish Guanylate Cyclase Type 3 Signaling in Cone Photoreceptors

**DOI:** 10.1371/journal.pone.0069656

**Published:** 2013-08-05

**Authors:** Ramona Fries, Alexander Scholten, Werner Säftel, Karl-Wilhelm Koch

**Affiliations:** 1 Department of Neuroscience, Biochemistry Group, Carl von Ossietzky University Oldenburg, Oldenburg, Germany; 2 Research Center Neurosensory Science, Carl von Ossietzky University Oldenburg, Oldenburg, Germany; University Zürich, Switzerland

## Abstract

The zebrafish guanylate cyclase type 3 (zGC3) is specifically expressed in cone cells. A specifc antibody directed against zGC3 revealed expression at the protein level at 3.5 dpf in outer and inner retinal layers, which increased in intensity between 3.5 and 7 dpf. This expression pattern differed from sections of the adult retina showing strong immunostaining in outer segments of double cones and short single cones, less intense immunoreactivity in long single cones, but no staining in the inner retina. Although transcription and protein expression levels of zGC3 are similar to that of the cyclase regulator guanylate cyclase-activating protein 3 (zGCAP3), we surprisingly found that zGCAP3 is present in a 28-fold molar excess over zGC3 in zebrafish retinae. Further, zGCAP3 was an efficient regulator of guanylate cyclases activity in native zebrafish retinal membrane preparations. Therefore, we investigated the physiological function of zGCAP3 by two different behavioral assays. Using the morpholino antisense technique, we knocked down expression of zGCAP3 and recorded the optokinetic and optomotor responses of morphants, control morphants, and wild type fish at 5–6 dpf. No significant differences in behavioral responses among wild type, morphants and control morphants were found, indicating that a loss of zGCAP3 has no consequences in primary visual processing in the larval retina despite its prominent expression pattern. Its physiological function is therefore compensated by other zGCAP isoforms.

## Introduction

Visual excitation and adaptation in vertebrate cone cells is much less understood than in rod cells [1]. Cones mediate photopic vision, allow discrimination of colors and are further able to maintain their responsiveness over 6–7 orders of magnitude of background light intensities. These remarkable performances require an efficient array of signaling molecules at the subcellular level.

In recent years zebrafish has become a favorite model organism for the study of cone phototransduction [2]. The zebrafish retina is equipped with one type of rod cell and four types of cone cells: short single (UV-sensitive) cones, long single cones (blue-sensitive) and double cones (green- and red-sensitive). Light responses of double cones and UV-sensitive cones were measured by suction-pipette recording in response to illumination [3], [4].

To explore the physiological function of photoreceptor-specific proteins in the zebrafish retina *in vivo*, a well-established method is the injection of antisense morpholino oligonucleotide probes into fertilized eggs [5], thereby preventing the translation of the target gene. Subsequent recording of the optokinetic reflex of the manipulated zebrafish larvae by applying visual stimuli allows understanding the *in vivo* function of the knockdown protein [6], [7]. However, this approach is restricted to a very narrow time frame during larval development, being optimal at 5–6 dpf. Although several key proteins of rod and cone phototransduction are functionally expressed at this time, their transcription and expression profiles can be different in the adult stage [8], [9].

However, progress in the genetic manipulation of zebrafish has revealed some crucial vision-related mechanism. For example, knockdown of the cone-specific opsin kinase GRK7 has a strong effect on photoresponse recovery and ectopic expression of GRK7 in zebrafish rods lowered the photosensitivity of rods [10], [11]; mutations in key proteins of the phototransduction cascade lead to blindness and cone degeneration [12].

Regulation of excitation and adaptation in photoreceptor cells depends strongly on the cytoplasmic Ca^2+^ concentration and on Ca^2+^ sensor proteins like recoverin, calmodulin and the guanylate cyclase-activating proteins (GCAPs) [13]–[15]. Zebrafish express a set of six GCAP isoforms, of which four are exclusively transcribed in cone cells (zGCAP3, 4, 5 and 7) [8], [16]. Isoforms of zGCAP differ in their Ca^2+^-sensing and Ca^2+^-activating properties [17]. A particular strong transcription in the larval state was observed for zGCAP3 [8] and investigations of its biochemical properties revealed that zGCAP3 is a strong activator of membrane sensory guanylate cyclases (GCs) sensing and mediating Ca^2+^-signals below 600 nM [9].

Putative targets of zGCAPs are three membrane bound GCs, namely zGC1, zGC2 and zGC3, encoded by the genes *gucy2f*, *gc2* and *gc3*, respectively. Transcription of zGC3 was cone specific and restricted to short single cones (SSC), long single cones (LSC) and double cones (DC), whereas transcription of zGC1 and 2 was only observed in rods and SSC [8]. A knockdown of the *gucy2f* gene leads to shortening of outer segments and visual impairment in the larvae as detected by the optomotor response assay [18]. A behavioral screen of mutagenized zebrafishes led to the identification of a mutant named *zatoichi*, which has defects in the *gc3* gene causing OKR and OMR impairment [19].

While these results indicate the importance of photoreceptor zebrafish GCs for normal cone vision, we still lack fundamental information about zGCs including expression at the protein level, relative abundance and regulatory aspects. To gain more insight into the operation of zGC3 in cone cells, the present work started on the following questions: what is the protein expression profile of zGC3 in larval and adult stages? What is the relative amount of zGC3 in the adult retina in comparison to the prominently expressed regulator zGCAP3? During progress of the work we further asked, how crucial the role of zGCAP3 is for the visual performance at the larval stage by using the morpholino anti-sense technique in combination with optokinetic and optomotor response recordings.

## Materials and Methods

### Zebrafish care

All experiments on zebrafish were performed in accordance with the European Communities Council Directive for animal use in science (86/609/EEC). Experiments were approved by Niedersächsisches Landesamt für Verbraucherschutz und Lebensmittelsicherheit, Oldenburg. Fish maintenance and care was performed as before [8], [9]. Zebrafish larvae were treated for immunohistochemistry as described in detail [9].

### Protein expression and purification

Proteins were heterologously expressed in *E. Coli* and purified by size-exclusion chromatography and/or anion-exchange chromatography as reported for zebrafish GCAPs [17], [20]. For the expression of myristoylated zGCAPs we used the A^6^S-mutant of zGCAP4 as described by Behnen et al. [20] and cloned a D^3^N-mutant of zGCAP5 using standard cloning procedures. The D^3^N point mutation in zGCAP5 was necessary to create a consensus sequence for yeast N-myristoyltransferase (NMT). For this purpose *E.coli* cells were cotransformed with the plasmid pBB-131 harbouring the gene for NMT from yeast *S.cerevisiae*. The purity of zGCAPs was verified by SDS-PAGE and was at least 90%. After purification proteins were dialyzed against NH_4_HCO_3_ buffer, concentrated, lyophilized and stored at −80°C until use.

### Polyclonal antibodies and immunoblotting

To obtain polyclonal antibodies directed against zGC3 we cloned the C-terminal part of zGC3 encompassing amino acids G1092-N1137 into a pGEX vector. A glutathione-S-transferase (GST) fusion protein was expressed in *E.coli*, purified on glutathione-affinity column and used for immunization of a rabbit. The corresponding serum was supplied from a company (Pineda-Antikörper Service, Berlin, Germany). To remove non-specific crossreactive antibodies from the serum it was further purified on an antigen-affinity column. For this purpose 450 µg of the antigen was coupled to CNBr-activated sepharose (GE healthcare, Munich, Germany) and the serum was passed over the column. After several washing steps in Tris-buffered saline, pH 7.4, the specific anti zGC3 antibody was eluted by 100 mM glycine pH 2.2 into 1M Tris-HCl pH 8.8.

Polyclonal antibodies directed against purified recombinant zGCAP5 were also produced by a company using rabbits for immunization (Pineda-Antikörper Service, Berlin, Germany). For detection of zGCAP3 we used the purified polyclonal anti-zGCAP3 antibody that was described previously [9]. The anti-GRK7 antibody directed against cone opsin kinase was a kind gift of Dr. Stephan Neuhauss at the University of Zurich, Switzerland. Immunoblotting and subsequent blot analysis was done exactly as described [9].

Relative affinities of the anti-zGC3 antibody for the antigen peptide and for the mature zGC3 in the retina were determined by a competition experiment. Increasing concentrations of antigen were preincubated with the antibody and then probed by immunoblotting against native zGC3 and the antigen-GST-fusion construct. Decrease of immunoreactive signals on blot membranes was quantified by determination of integrated density values on an AlphaImager (Biozyme, Germany) and the antigen amount causing halfmaximal decrease of signals was compared.

### Immunohistochemistry

Immunolocalization of zGC3 expression in larval and adult retina slices was done exactly as previously described for zGCAP3 [9] (see also below). The dilution of the first antibody was 1∶400, the secondary antibody was a goat anti-rabbit IgG coupled to alkaline phosphatase (Jackson Dianova, USA) and diluted to 1∶200.

### Guanylate cyclase assay

Guanylate cyclase activity and activation properties of cone-specific zGCAPs were measured as described before under very dim red light [17]. As a source of sensory zGCs we used washed membranes of zebrafish retinae as previously described for the analysis of zGCAP4 [20]. ZGCAPs were incubated at a concentration of 10 µM with retinal membranes in buffer with the following final concentrations: 40 mM Hepes, pH 7.4, 56 mM KCl, 2.5 mM NaCl, 10 mM MgCl_2_, 2 mM GTP, and 0.04 mM ATP, 0.1 mM zaprinast. Quantitative analysis was done as before [17].

### Morpholino antisense knockdown

In order to evaluate the impact of zGCAP3 on larval cone vision a morpholino oligonucleotide antisense probe was designed to cover the start codon and a stretch of the 5′-untranslated region of the *guca1c* gene, to prevent expression of the gene product. Morpholino antisense oligonucleotides (MOs) were synthesized by a commercial supplier (GENE TOOLS, LLC Philomath, OR 79370 USA) and stored at −20°C until use. A morpholino was designed to bind at the 5′-UTR of the zGCAP3 mRNA and the sequence of morpholino zGCAP3.1 (3′-CTTTCCGAAGCCTCCTCAATACCCG-5′) covered the startcodon. For control injections a standard control morpholino was used (3′-ATATTTAACATTGACTCCATTCTCC-5′). Stock solutions of MOs (1 or 2 mM) were prepared by dissolving them in Danieaús solution (5 mM Hepes pH 7.6, 58 mM NaCl, 0.7 mM KCl, 0.4 mM MgSO_4_, 0.6 mM Ca(NO_3_)_2_) supplemented with 0.1% phenol red and stored at −20°C. MO solutions (2 nL) were injected into the yolk of fertilized eggs at the 1 to 2 cell stage. Eggs were previously washed in 30% Danieaús solution and kept in the same solution after injection. Vitality of developing larvae was controlled every day and compared with WT and control injected larvae. Different concentrations of MOs were tested to find out the optimal concentration for a knockdown of zGCAP3 without causing nonspecific malformation. Larvae at 5 and/or 6 dpf were tested by immunoblotting and immunohistochemistry. Ten eyes of larvae at 5 or 6 dpf were homogenized in 4 µl of bidistilled H_2_O, centrifuged at 14,000×g and the supernatant was supplemented with 20 mM Na-phosphate buffer pH 7.4, 1 mM EGTA and boiled for 5 min at 95°C before electrophoresis and blotting. Immunohistochemistry was exactly as described [9].

### Optokinetic response measurements

OKRs were recorded on MO treated larvae at 5 dpf. Single larvae were immobilized in 6% methylcellulose and moving stimuli were presented on surrounding computer LCD screens. Movement of the eyes was monitored by a high-resolution camera. Details of the OKR setup and monitor system are described elsewhere [21]. The stimuli (moving linear gratings) were presented for 4 seconds in one direction (50 deg/sec) before the direction was reversed. After 7 changes of direction the stimulus was stopped. Saccadic eye movements were observed and evaluated according to Brockerhoff et al. [22]. In order to test for visual acuity stimuli of different sizes (spatial frequency of 0.04–0.2 cycles/deg) were employed (see also [23]).

### Optomotor response

The optomotor response of zebrafish is a reflex to stabilize the body position in a moving environment [24]. Optomotor response measurements were done exactly as described by Striebel-Kalish et al. [18] using a moving grating as stimulus that was presented by a computer program freely available (MovingGrating Vs 1.2; http://michaelbah.de/stim/). Coloured stimuli were created by using either green (maximum of transmission at 525 nm) or red (maximum transmission starting at 600 nm) filtering transparencies. The light intensity of the computer screen was always adjusted to the same intensity level.

### Statistical analysis

Larvae that were used in behavioral studies were grouped in three populations, morpholino-treated, control morpholino-treated and wildtype (WT) and results from these studies were statistically analyzed with a Students *t*-test according to ref. [25].

## Results

### Subcellular localization of zGC3 in larval and adult retinae

**Figure 1:**
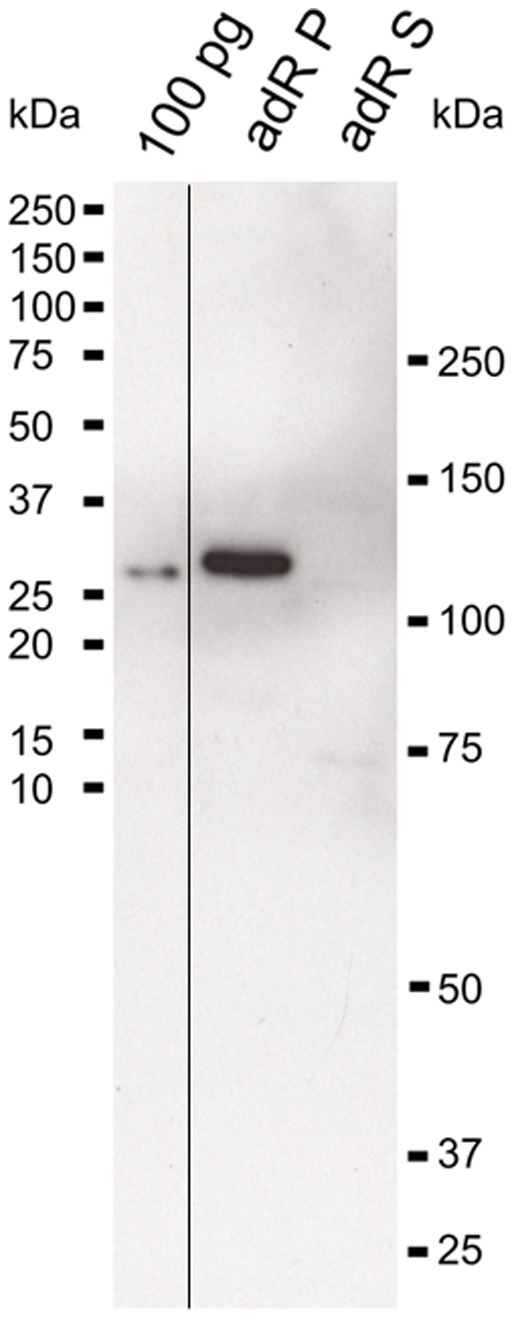
Specificity of anti-zGC3 antibody. Hundred pg of the antigen, the pellet adRP) and soluble fraction (adRS) of 1/10 of an adult zebrafish retina each were probed with the purified polyclonal anti-zGC3 antibody (1∶1000); secondary antibody was a goat anti rabbit coupled to peroxidase (1∶5000).

**Figure 2:**
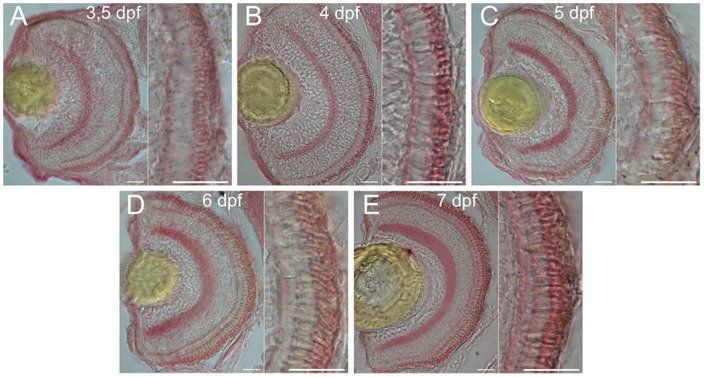
Temporal expression profile of zGC3 during larval development. Cryosections of larval eyes were probed with the anti-zGC3 antibody (1∶400) at 3.5 – 7 dpf (A–E) and stained with Fast Red. Left part of each panel is a section through the whole eye, right part shows the photoreceptor layer. Scale bar  = 20 µm.

**Figure 3:**
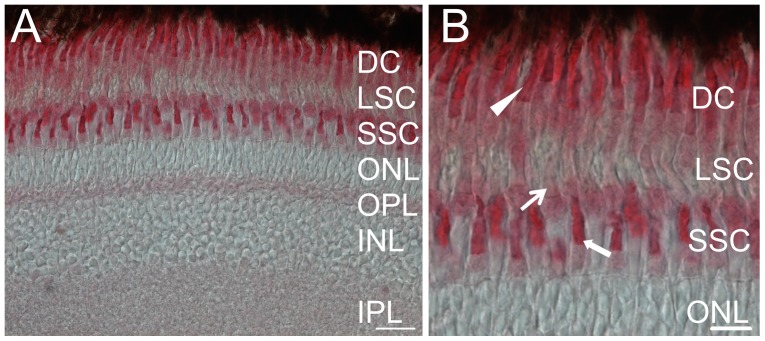
Immunostaining of zGC3 in cryosections of adult zebrafish retina. Sectionas were probed with the anti-zGC3 antibody (1∶400). The secondary antibody was used at 1∶200 dilution. (A) Section of a whole retina, cell layers are indicated as double cones (DC), long single cones (LSC), short single cones (SSC), outer nuclear layer (ONL), outer plexiform layer (OPL), inner nuclear layer (INL) and inner plexiform layer (IPL). (B) Magnified part of (A). DC: closed arrowhead; LSC: thin arrow; SSC: bold arrow. Scale bar  = 20 µm in (A) and 10 µM in (B).

The purified polyclonal antibody against zGC3 recognized a protein band of ca. 120 kDa in a retina membrane homogenate after western blotting (Figure 1). No corresponding band was detected in the fraction of soluble retina proteins (Figure 1, lane labeled adR S), nor was any other band recognized by the antibody. The fusion protein that was used for immunization of the rabbit was labeled already at an amount of 100 pg (Figure 1, please note different scaling of lanes in the blot). Thus, the antibody proved to be highly specific for detecting a band with the expected molecular mass of 127.3 kDa for zGC3. The antibody was further used to monitor the spatial-temporal expression pattern of zGC3 in the larval retina from 3.5 dpf to 7 dpf (Figure 2A–E). Distinct labeling of the photoreceptor cell layer was seen throughout the whole retina and increased slightly in intensity during maturation. In addition the plexiform layers were labeled, but no staining was observed in the nuclear layers. This expression pattern became more differentiated in the adult retina, where a strong staining was observed in the outer segments of DCs and SSCs (Figure 3A and B). LSCs showed only weak labeling of outer segments, but slightly stronger staining of inner segments. However, the significant labeling of the plexiform layers seen in the larval retina almost disappeared in the section from the adult retina (Figure 3A).

### Relative amount of zGC3 in adult retina and ratio to zGCAP3

**Figure 4:**
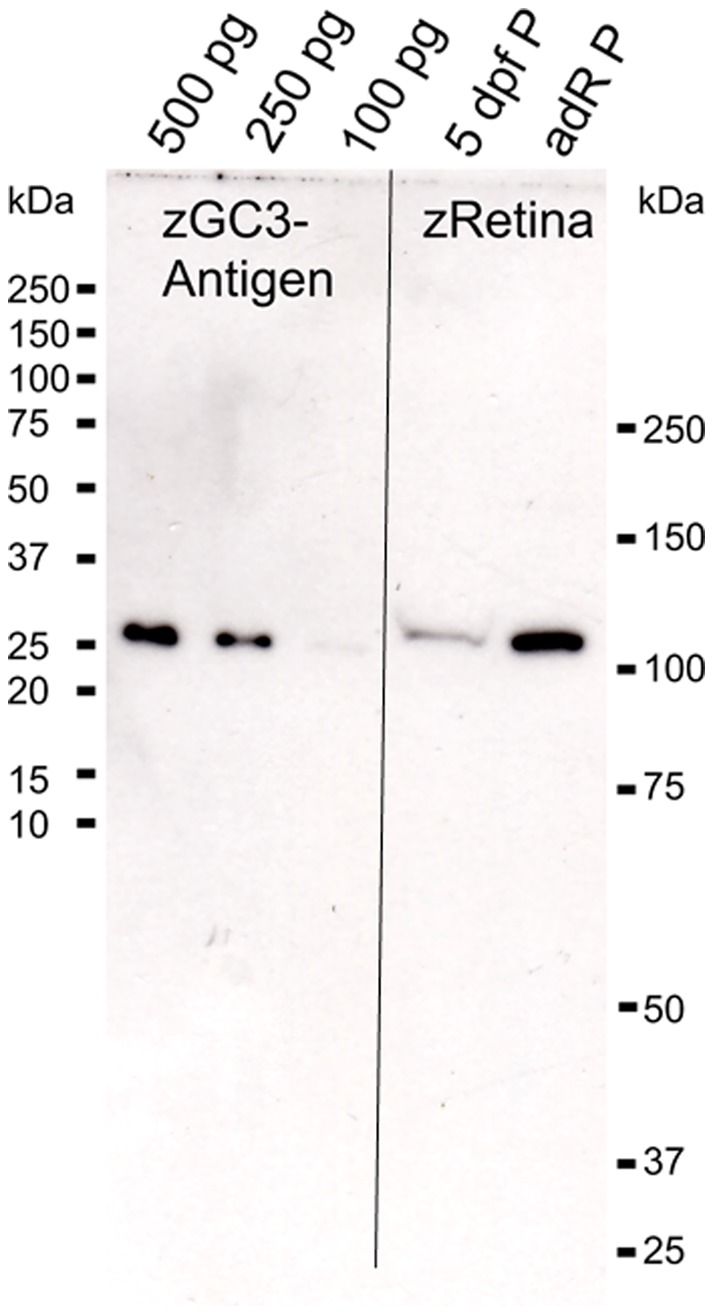
Quantitative estimation of zGC3 in adult zebrafish retina by western blotting. Different amounts of zGC3 antigen (100 pg, 250 pg and 500 pg) were used as calibration standards and compared with the pellet fraction of 5 dpf old larva eyes (10 eyes were used for homogenization) and of 1/10 of an adult retina (adRP).

In mammalian photoreceptors GCs and GCAPs are present at almost equimolar ratios [26]. We expected to find similar equal molar ratios of zGC3 to zGCAP3, since the intensity of photoreceptor staining by the anti-zGC3 antibody is comparable to our recent results we described for the immunolocalization of zGCAP3 in the adult zebrafish retina [9]. Employing quantitative western blotting we determined an amount of 3.8 ng zGC3 or 3×10^−14^ mole per adult retina (Figure 4). In our recent study we obtained 9 ng of zGCAP3 or 4×10^−13^ mole per adult retina [9], which would then give a molar ratio for zGC3 to zGCAP3 of 1∶14 or 1∶28 by assuming that the functional unit of the zGC3 is a dimer similar to mammalian sensory GCs [27]. For the quantification procedure it is of crucial importance that the anti-zGC3 antibody has nearly the same affinities for the antigen used for raising the antibody and for the mature protein expressed in the retina. We tested the relative affinities by a competition experiment using increasing concentrations of the antigen, which is preincubated with the antibody and then probed with zebrafish membranes and the antigen by western blotting (Figure S2). The affinities of the antibody for native zGC3 in retinal membranes was slightly lower (about two-fold) than for the GST-fusion construct. However, this difference could not account for the large difference that we determined for the expression of zGCAP3 and zGC3. Thus, the surprising result of a large molar excess of zGCAP3 to zGC3 raised the question whether the abundant expression of zGCAP3 is reflected in the activating properties. Therefore, it prompted us to compare the activating properties of cone specific zGCAPs when reconstituted with native zebrafish membranes containing zGCs. Previous comparable measurements on zGCAP function employed mammalian GCs as a reliable assay system due to the limitation in zebrafish retinal material [17].

### Activity profiles of zGCs in zebrafish membranes

**Figure 5:**
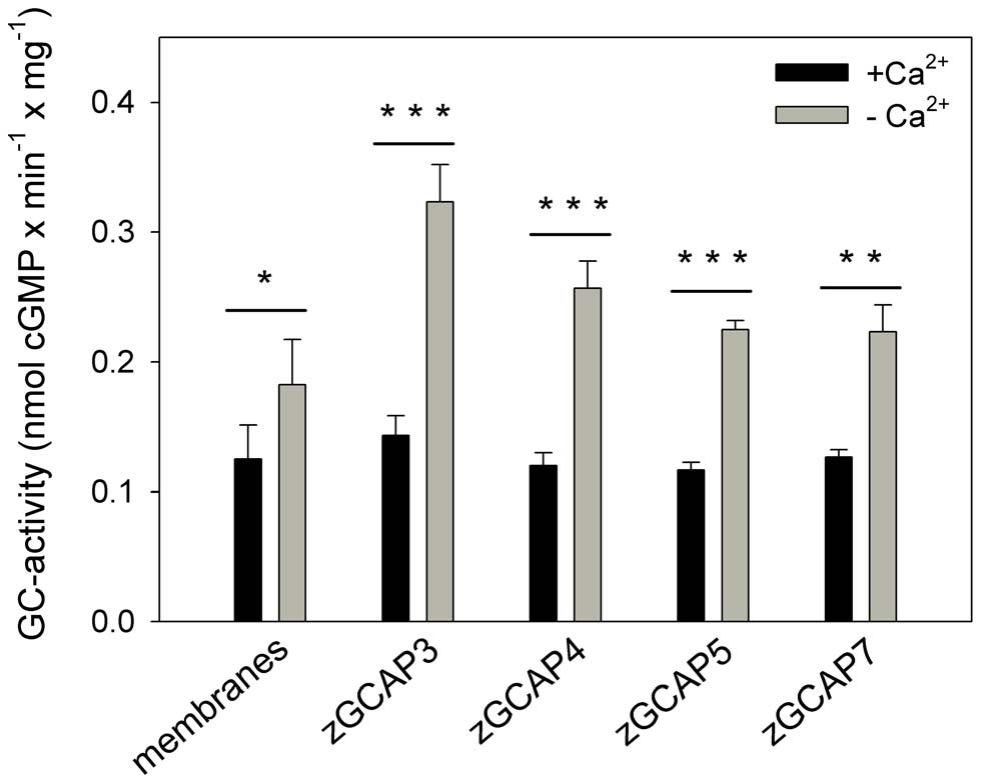
GC activities in adult zebrafish retinae. Membranes from adult zebrafish retinae were incubated with 10 µM recombinant myristoylated zGCAPs as indicated. GC activities were measured in the presence of 100 µM Ca^2+^ (+ Ca^2+^) or in the absence of Ca^2+^ (2 mM EGTA, -Ca^2+^). Data are mean± s.d. of three replicates. Differences of activities in the presence of zGCAPs were highly significant: zGCAP3 (*t* = 9.53, ***P≤0.001); zGCAP4 (*t* = 10.26, ***P≤0.001); zGCAP5 (*t* = 17.9, ***P≤0.001); zGCAP7 (*t* = 7.76, **P≤0.01); membranes (*t* = 2.27, *P<0.05).

The Ca^2+^-dependent activation of zGCs in retina membrane preparations were measured for zGCAP3, 4, 5 and 7. For this purpose each purified recombinant zGCAP was reconstituted with a membrane suspension and incubated at high (100 µM Ca^2+^) and low Ca^2+^-concentrations (2 mM EGTA) yielding the results shown in Figure 5. The highest activities were measured with zGCAP3 indicating that zGCAP3 is indeed a major activator of zGCs in native zebrafish retinae.

An estimation of GC activities in zebrafish retinae yielded approximately 0.1–0.4 nmol cGMP×min^−1^ per mg protein (Figure 5; ref. [20]), which is similar to membrane bound GC activities in mammalian [26], [28].

### Knockdown of zGCAP3 by morpholino antisense probes

**Figure 6:**
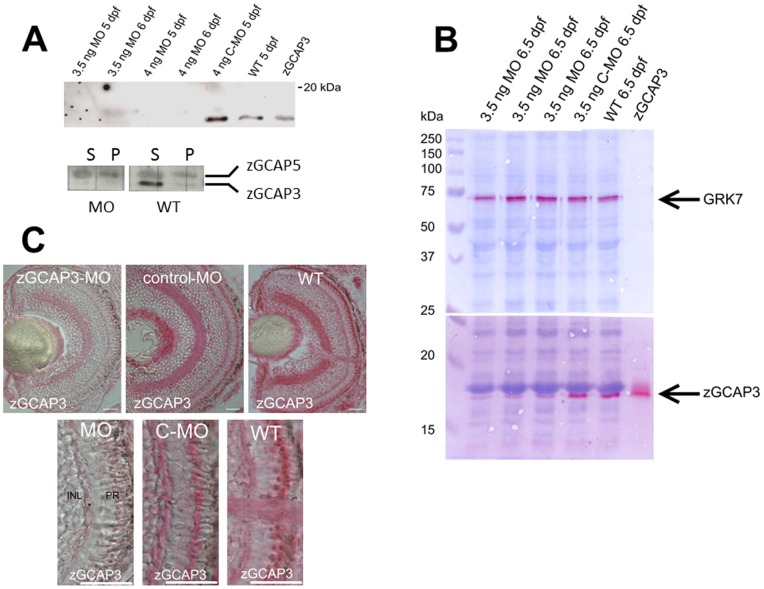
Morpholino knockdown of zGCAP3 in zebrafish larvae. (A) upper panel: Immunoblotting of homogenized eyes obtained from larvae at 5 and 6 dpf that had been injected with the indicated amount of MOs. Recombinant nonmyristoylated zGCAP3 (1 ng) was loaded on the very right lane. Purified polyclonal anti-zGCAP3 was used at 1∶250, second antibody was a peroxidase-coupled goat anti-rabbit IgG at a dilution of 1∶5000. Lower panel: Immunoblotting as above of MO treated and WT larvae (5 dpf, 10 eyes each). Supernatant (S) and pellet (P) fraction of larval eye homogenates were probed by polyclonal anti-zGCAP3 and anti-zGCAP5 antibodies (dilution of 1∶200 and 1∶250, respectively). Second antibody was a peroxidase-coupled goat anti-rabbit IgG at a dilution of 1∶2500. (B) Analysis of larval eyes at 6.5 dpf by immunoblotting. The same larvae (three lanes that were identically labeled “MO 6.5 dpf”) had been investigated in the optomotor response measurements at 6 dpf. Proteins were first fixed in the polyacrylamide gel according to ref. [34] before transfer to the blot membrane. Reactive antibody binding was visualized by using Fast Red. The anti-GRK7 antibody was used at a dilution of 1∶1000. Use of other antibodies as in (A). (C) Immunohistochemistry of larval eyes at 5 dpf as indicated. Cryosection of 10 µm were labeled with the purified polyclonal anti-zGCAP3 antibody at a dilution of 1∶2000, secondary antibody was a goat anti-rabbit conjugated to alkaline phosphatase, dilution at 1∶200. Scale bars: 20 µm. The lower part of the figure is a magnified part of the upper figures showing the photoreceptor cell layer.

In order to evaluate the impact of zGCAP3 on larval cone vision a morpholino oligonucleotide antisense probe was designed to cover the start codon and a stretch of the 5′-untranslated region of the *guca1c* gene, to prevent expression of the gene product. Injection of 3.5–4.0 ng of the antisense probe did not impair the normal larval development. Expression of the protein product zGCAP3 is already detectable at 3 dpf onward [9]. Application of the morpholino antisense probe prevented the translation of the *guca1c* gene (Figure 6A), since protein extracts from WT larval eyes at stages 5 and 6 dpf were probed by immunoblotting and contained zGCAP3, whereas samples of the morphant larvae showed no zGCAP3 expression. Injection of a control morpholino probe left the expression of zGCAP3 intact (Figure 6A, lane labelled C-MO).

Recovery of zGCAP3 expression started at 6.5 dpf, which can be seen in the double-staining of the blot membrane in Figure 6B. While strong expression was seen in the WT and after injection of the control morpholino (bands stained by Fast Red), a faint red band became visible after 6.5 dpf. No change in the expression pattern was observed for the cone-specific kinase GRK7 (red bands in the upper part of the blot in Figure 6B). Another cone specific GCAP, zGCAP5 for example, was neither up- nor downregulated (Figure 6A, lower panel).

Immunohistochemistry on larval retina sections revealed the same picture showing a complete knockdown of zGCAP3 at 5 dpf, while WT and larvae that were injected with the control morpholino showed the expression pattern of zGCAP3 (Figure 6C) known from a previous report [9]. Control staining of retina sections with the anti-GRK7 antibody revealed the normal expression of GRK7 under all conditions similar as in Rinner et al [10] (data not shown).

### Optokinetic and optomotor response measurements

**Figure 7:**
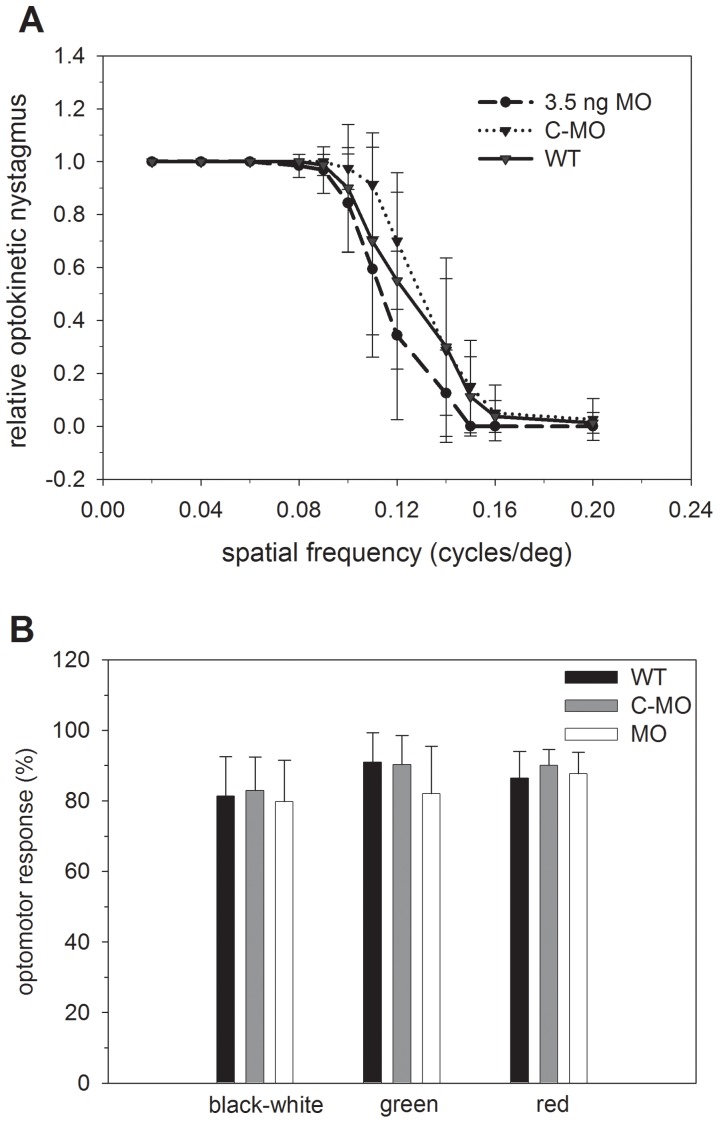
Visual behavioral assays. (A) Optokinetic response measurements of WT, MO and control MO larvae: 8–10 larvae at 5 dpf were investigated by presenting stimuli of a pattern of black and white stripes as described in the Methods. Results of black and green, black and red, and black and blue striped pattern are shown in supplementary Fig. S1. The relative optokinetic nystagmus is shown as a function of the spatial frequency (mean±s.d.). (B) Optomotor response evaluation of larvae as indicated at 6 dpf. For the black and white stimulus the mean±s.d. of 52 measurements is shown (MO versus WT: *t* = 0.98, *P*≥0.05; MO versus control MO: *t* = 2.0, *P* = 0.05; control MO versus WT: *t* = 1.05, *P*≥0.05), for the coloured stimuli the mean±s.d. of 12 data sets each is shown (black/green MO versus WT: *t* = 1.94, *P* ≥ 0.05; MO versus control MO: *t* = 1.8, *P*≥0.05; control MO versus WT: *t* = 0.19, *P*≥0.05). Black / red: MO versus WT: *t* = 0.44, *P*≥0.05; MO versus control MO: *t* = 1.05, *P*≥0.05; control MO versus WT: *t* = 1.4, *P*≥0.05).

Since the expression of zGCAP3 was completely abolished, we next tested in two visual behavior assays, how this knockdown affected the normal vision of the manipulated larva. Optokinetic response (OKR) measurements are particularly useful in the assessment of visual deficits. A moving pattern of black and white or black and colored (green, red and blue) stripes was presented to the fish by surrounding computer screens. All larvae exhibited the typical saccadic eye movements observed with WT. The optokinetic nystagmus decreased with increasing spatial frequency (Figure 7A and S1) and data sets showed large overlapping standard deviations. The significance level was for most spatial frequencies *P>*0.05 except for spatial frequencies 0.11, 0.12 and 0.15 the OKR of the control MO larvae was slightly higher than for MO larvae (*P*<0.05) (Figure 7A). The OKR of WT larvae was only higher than the OKR of MO larvae at a spatial frequency of 0.15 (*P*<0.05). In OKR tests employing colored stripes we found no significant differences (*P*>0.05) between responses of WT, morphants and control morphants (Figure S1), but in two cases the OKR of control MO was higher than that of WT larvae (*P*<0.05 for blue/black pattern at spatial frequency of 0.11) and MO larvae (*P*<0.05 for red/black pattern at spatial frequency of 0.12), respectively. The green/black pattern caused higher OKR of MO larvae than WT larvae at a spatial frequency of 0.15 (*P*<0.05) (Figure S1). Taking together the OKR of MO larvae did not differ from that of WT larvae in a significant manner.

Optomotor responses of zebrafishes were monitored by presenting a moving grating to groups of 15 fishes and counting the number of fishes found in the target region of the tank after 90 seconds. Red and green cones provide the dominant input for the optomotor response in larvae [24]. Groups of WT, morphants and control morphants were tested. On average 80–90% of all fishes were moving normally to the target region indicating that a zGCAP3 knockdown had no consequence for the optomotor response behavior (Figure 7B). It also made no significant difference, whether the grating consisted of alternating black and white or black and green/red stripes (*P*≥0.05).

These results indicated that a knockdown of zGCAP3 in larval eyes has not led to a loss of photoreceptor function. Instead, absence of zGCAP3 was very likely compensated by the presence and operation of other zGCAP forms that have similar activating properties (Figure 5; ref. [17]).

## Discussion

Zebrafish cone cells express six different GCAP isoforms that are expected to regulate three different sensory GCs [8], [16], [29]. We started our present study to gain insight into the spatial-temporal expression of the cone specific guanylate cyclase zGC3 and its relative amount in adult retinae. The protein was prominently labeled in DC and SSC, but appeared less abundant in LSC. Thus, expression at the protein level was in general agreement with our previous detection of transcription by in situ hybridization, which indicated that zGC3 is a cone specific cyclase. Due to the strong immunolabelling by the anti-zGC3 antibody we expected an almost equal molar ratio to zGCAP3, for which we recently observed a similar strong staining of photoreceptor cone cells. Furthermore, zGC3 and zGCAP3 parallel each other in several aspects: their transcripts were detected already at 3 dpf in the developing retina coinciding with the onset of visual function [29] and expression at the protein level was first visible at 3.25–3.5 dpf and became stronger in the following days (see ref. [9] for zGCAP3 and Figure 2A–E for zGC3). In addition, biochemical studies on zGCAP3 showed that it is a strong activator of membrane GCs exhibiting a high sensitivity for Ca^2+^ [9], [17].

However, several findings of the present study were different from our expectations. We detected a large 28-fold molar excess of zGCAP3 over zGC3 instead of almost equimolar ratios of GCs to GCAPs known from mammalian [26] and even carp retinae [30]. This could indicate that zGCAP3 might have targets in addition to zGC3, a suggestion that is supported by our immunohistochemical staining of zGCAP3 in adult retinae [9]. For example, intense immunostaining by the anti-zGC3 antibody was restricted to the outer segments of DC and SSC (Figure 3), whereas staining with the anti-zGCAP3 antibody was stronger for cone inner segments and the outer plexiform layer. Immunoreactivity against zGCAP3 was even observed in the inner plexiform and ganglion cell layer [9], but these layers showed no immunoreactivity against the anti-zGC3 antibody.

Collectively these results hint to a prominent role for zGCAP3 in primary visual processes and also make it a premium candidate to test for its physiological role in a behavioral assay by comparing WT and morphant larvae. Using the morpholino antisense technique we could knockdown the expression of the zGCAP3 at 5 and 6 dpf (Figure 6), but the optokinetic and optomotor responses of the morphants were indistinguishable from the WT and control recordings. In more detail, the comparison of WT, control MO and MO revealed no significant differences in OMR (*P*>0.05) and only small differences in OKR (*P*<0.05) for some spatial frequencies, but only for seven out of 156 cases and without a clear tendency (see Figure 7A and S1). Thus, we think these very minor differences do not reflect an impairment of visual processing, when the expression of zGCAP3 is prevented. Two explanations are conceivable for these findings. First, although zGCAP3 is normally expressed in cone cells at 3.5 dpf onwards, it might not be functional in WT larvae. For example, it lacks a covalently attached myristoyl group in the early larval state and the switch to the myristoylated form occurs later than 7 dpf [9]. We do not consider this as very likely, because nonmyristoylated zGCAP3 is functional in *in vitro* studies. Second, the presence of other zGCAPs could compensate the lack of zGCAP3. All zGCAPs can activate zebrafish GCs in a Ca^2+^-dependent manner (Figure 5) and although their Ca^2+^-operation range and their maximal stimulatory activities are not identical [17] their activity profiles match to prevent a complete loss of visual function in MO larvae (Figure 5). Further, we have no indication that other zGCAPs are up-regulated (Figure 6A). Instead, we think that one or more zGCAP isoforms would step into regulation, when zGCAP3 is lacking. We consider this as a reasonable assumption, because cones are not differentiated to their final stage at 6 dpf and proteins are not restricted in their spatial expression as in the adult fish, which facilitates compensation of protein function by isoforms. A good substitute for zGCAP3 might be zGCAP4, because its transcripts are also detected at 3.5 dpf [8] and it is a strong activator of membrane bound GCs with a similar apparent affinity for GCs [17], [20]. In addition, zGCAP3 might be a regulator of a different target that is not involved in primary visual processes and is expressed in photoreceptor inner segments or in other retinal layers.

Finally, membrane bound GC activities in zebrafish adult retinae were similar to GC activities in mammalian retinae, but were thus about four-fold lower than the high synthetic activity reported for carp cones [30]. We cannot exclude that the *in vitro* assay conditions allow only to measure sub-optimal activity values, although our assay system gives reproducible values for mammalian GC activities (see for example ref. [31], [32]). The low cGMP synthesis rate could however, reflect general low cytoplasmic cGMP levels in zebrafish cones. One consequence of such dark cGMP levels would be a faster hydrolysis rate after illumination causing the fast rising phase kinetics of zebrafish cone photoresponses [33].

## Supporting Information

Figure S1 **Visual behavioral assays.** Optokinetic response measurements of WT, MO and control MO larvae: 8–10 larvae at 5 dpf were investigated by presenting stimuli of a pattern of black and green (upper part), black and red (middle part), and black and blue (lower part) striped pattern. The relative optokinetic nystagmus is shown as a function of the spatial frequency (mean ± s.d.).
(DOCX)

Figure S2 **Competition assay for determining the relative affinities of the anti-zGC3 antibody.** Zebrafish membranes and the antigen-GST-fusion construct were electrophoresed and blotted. The anti-zGC3 antibody was preincubated with increasing amounts of antigen and then probed as indicated. Immunoblot reactivities were evaluated as integrated density values (IDV)
(DOCX)
